# Upregulation of PD-L1 Expression by Prostaglandin E_2_ and the Enhancement of IFN-γ by Anti-PD-L1 Antibody Combined With a COX-2 Inhibitor in *Mycoplasma bovis* Infection

**DOI:** 10.3389/fvets.2020.00012

**Published:** 2020-02-20

**Authors:** Shinya Goto, Satoru Konnai, Yuki Hirano, Junko Kohara, Tomohiro Okagawa, Naoya Maekawa, Yamato Sajiki, Kei Watari, Erina Minato, Atsuhi Kobayashi, Satoshi Gondaira, Hidetoshi Higuchi, Masateru Koiwa, Motoshi Tajima, Eiji Taguchi, Ryoko Uemura, Shinji Yamada, Mika K. Kaneko, Yukinari Kato, Keiichi Yamamoto, Mikihiro Toda, Yasuhiko Suzuki, Shiro Murata, Kazuhiko Ohashi

**Affiliations:** ^1^Department of Disease Control, Faculty of Veterinary Medicine, Hokkaido University, Sapporo, Japan; ^2^Department of Advanced Pharmaceutics, Faculty of Veterinary Medicine, Hokkaido University, Sapporo, Japan; ^3^Agriculture Research Department, Animal Research Center, Hokkaido Research Organization, Shintoku, Japan; ^4^Department of Veterinary Clinical Medicine, Faculty of Veterinary Medicine, Hokkaido University, Sapporo, Japan; ^5^School of Veterinary Medicine, Rakuno Gakuen University, Ebetsu, Japan; ^6^Shibetsu Animal Hospital, Shibetsu, Japan; ^7^Department of Veterinary Medical Science, Faculty of Agriculture, University of Miyazaki, Miyazaki, Japan; ^8^Department of Antibody Drug Development, Graduate School of Medicine, Tohoku University, Sendai, Japan; ^9^New Industry Creation Hatchery Center, Tohoku University, Sendai, Japan; ^10^Research and Development Center, Fuso Pharmaceutical Industries, Ltd., Osaka, Japan; ^11^New Business and International Business Development, Fuso Pharmaceutical Industries, Ltd., Osaka, Japan; ^12^Division of Bioresources, Research Center for Zoonosis Control, Hokkaido University, Sapporo, Japan; ^13^Global Station for Zoonosis Control, Global Institution for Collaborative Research and Education (GI-CoRE), Hokkaido University, Sapporo, Japan

**Keywords:** immunoinhibitory molecules, PD-1, PD-L1, T-cell exhaustion, prostaglandin E_2_, immune dysfunction, *Mycoplasma bovis*, cattle

## Abstract

Bovine mycoplasmosis caused by *Mycoplasma bovis* results in pneumonia and mastitis in cattle. We previously demonstrated that the programmed death 1 (PD-1)/PD-ligand 1 (PD-L1) pathway is involved in immune dysfunction during *M. bovis* infection and that prostaglandin E_2_ (PGE_2_) suppressed immune responses and upregulated PD-L1 expression in Johne's disease, a bacterial infection in cattle. In this study, we investigated the role of PGE_2_ in immune dysfunction and the relationship between PGE_2_ and the PD-1/PD-L1 pathway in *M. bovis* infection. *In vitro* stimulation with *M. bovis* upregulated the expressions of PGE_2_ and PD-L1 presumably via Toll-like receptor 2 in bovine peripheral blood mononuclear cells (PBMCs). PGE_2_ levels of peripheral blood in infected cattle were significantly increased compared with those in uninfected cattle. Remarkably, plasma PGE_2_ levels were positively correlated with the proportions of PD-L1^+^ monocytes in *M. bovis*-infected cattle. Additionally, plasma PGE_2_ production in infected cattle was negatively correlated with *M. bovis*-specific interferon (IFN)-γ production from PBMCs. These results suggest that PGE_2_ could be one of the inducers of PD-L1 expression and could be involved in immunosuppression during *M. bovis* infection. *In vitro* blockade assays using anti-bovine PD-L1 antibody and a cyclooxygenase 2 inhibitor significantly upregulated the *M. bovis*-specific IFN-γ response. Our study findings might contribute to the development of novel therapeutic strategies for bovine mycoplasmosis that target PGE_2_ and the PD-1/PD-L1 pathway.

## Introduction

Bovine mycoplasmosis caused by *Mycoplasma bovis* is characterized by chronic pneumonia, therapy-resistant mastitis, otitis, and arthritis (1–4). *M. bovis* has several immunosuppressive characteristics *in vitro*, such as inhibition of proliferative response of bovine peripheral blood mononuclear cells (PBMCs), induction of apoptosis in bovine lymphocytes, and delay of apoptosis in bovine monocytes, along with suppressed production of interferon (IFN)-γ and tumor necrosis factor (TNF)-α (5–7). These characteristics could cause chronic progression of the disease and, especially during the lung infection, and allow coinfection with other bacteria and viruses (2, 3). However, the mechanisms underlying the dysfunction in *M. bovis* infection have remained unclear.

Programmed death-1 (PD-1) is an immunoinhibitory receptor that is expressed on activated T cells and has been involved in immune dysfunction during various chronic infections (8–10). After binding of PD-ligand 1 (PD-L1), PD-1 induces T-cell dysfunction by inhibiting T-cell receptor signaling. This immune dysfunction is called T cell exhaustion. On the other hand, treatment with monoclonal antibodies (mAbs) specific to PD-1 or PD-L1 is capable of reactivating functions of exhausted T cells. PD-1/PD-L1 could be a potential therapeutic target in patients with chronic infections. We previously demonstrated that the expression of PD-1 on T cells and PD-L1 on monocytes were significantly increased in *M. bovis*-infected cattle (11). Furthermore, the blockade of PD-1/PD-L1 pathway by antibodies *in vitro* activated immune responses in *M. bovis*-infected cattle (11). Therefore, the T cell exhaustion caused by PD-1/PD-L1 might be involved in the immune dysfunction during bovine mycoplasma.

Prostaglandin E_2_ (PGE_2_) is one of the lipid mediators that are derived from arachidonic acid synthesized by cyclooxygenase isoenzymes (COX-1 and COX-2) (12, 13). It is known that PGE_2_ promotes the immune dysfunction associated with several tumors and chronic inflammation (14, 15). Interestingly, recent reports on cancer have shown a relationship between PGE_2_ and the PD-1/PD-L1 pathway (16, 17). Our previous study demonstrated that PGE_2_ suppressed the Th1 response in cattle and upregulated PD-L1 expression in bovine PBMCs *in vitro* (18). In addition, PGE_2_ showed immune dysfunction effects in other bovine chronic diseases, Johne's disease (18), which is known to be a chronic bovine disease by *Mycobacterium avium* subsp. *paratuberculosis* (MAP) and bovine leukemia virus (BLV) infection (19). Furthermore, the dual blocking of PGE_2_ and the PD-1/PD-L1 pathway substantially enhanced the MAP and BLV-specific T-cell reaction in cattle. However, the involvement of PGE_2_ in the immune dysfunction of bovine mycoplasmosis has not yet been fully investigated.

In this study, we investigated the role of PGE_2_ in immune dysfunction and the relationship between PGE_2_ and the PD-1/PD-L1 pathway in *M. bovis* infection. We believe that our findings will help in the development of novel strategies for bovine mycoplasmosis.

## Materials and Methods

### Bacterial Strain

*M. bovis* strain PG45 (ATCC25523) was used in the experiments of this study. *M. bovis* was cultured in NK broth (Miyarisan Pharmaceutical, Tokyo, Japan) at 37°C for 72 h and collected by centrifugation. The bacteria were washed with phosphate-buffered saline (PBS), and colony-forming units were counted using the NK agar plate (Miyarisan Pharmaceutical) by dilution method. The bacteria were then resuspended in RPMI 1640 medium (Sigma-Aldrich, St. Louis, MO, USA) containing 10% heat-inactivated fetal bovine serum (FBS) (Thermo Fisher Scientific, Waltham, MA, USA), 2 mM L-glutamine, 100 U/ml penicillin, and 100 μg/ml streptomycin (Thermo Fisher Scientific) and stored at −80°C until use.

### Ethical Approval

All experimental procedures were conducted following approval from the local committee for animal studies according to the Hokkaido University (17–24). Written informed consent was obtained from all owners of cattle sampled in this study.

### Bovine Samples

Peripheral blood samples of cattle were obtained from adult Holstein-breed cattle in Hokkaido, Japan. Cattle infected with *M. bovis* were diagnosed clinically and microbiologically at Rakuno Gakuen University and Hokkaido University. *M. bovis* infection was confirmed with PCR by using clinical samples as described previously (21). The symptoms of infected cattle included pneumonia, arthritis, and otitis media. Control blood samples of *M. bovis*-uninfected cattle were obtained from adult Holstein-breed cattle in Hokkaido, Japan. Negative control cattle were serologically negative for *M. bovis* infection according to enzyme-linked immunosorbent assay (ELISA). Briefly, ELISA plates (Thermo Fisher Scientific) were coated with 100 μl of solubilized *M. bovis* (PG45, 50 μg/ml in carbonate buffer) as the target antigen at 37°C for 17 h. After washing the plates four times with a wash solution (PBS with 0.1% Tween20), 100 μl of serum sample was added to each plate. After incubation at 37°C for 1 h, the plates were washed three times with TSB-T [PBS with 50 mM Tris, 0.1% bovine serum albumin (BSA), and 0.05% Tween20] and incubated with skim milk (Wako, Osaka, Japan) as a protein blocker at 37°C for 2 h. After washing the plates thrice with TSB-T, protein G-conjugated horseradish peroxidase (Rockland Immunochemicals, Pottstown, PA, USA) was added to the wells and the plates were incubated at 37°C for 1 h. After washing the plates thrice with TSB-T, 3-ethylbenzothiazolin-6-sulfonic acid (ABTS; Sera Care, Milford, MA, USA) was added to the wells and the optical density was measured at 415 nm using a plate reader (iMarkTM Microplate Absorbance Reader, Bio-Rad, Hercules, CA, USA).

### Cell Preparation and Culture

Bovine PBMCs were purified from blood samples with density gradient centrifugation using Percoll (GE Healthcare, Little Chalfont, England, UK). As described previously (20), CD14^+^ cells were freshly isolated from bovine PBMCs using the autoMACS Pro System (Miltenyi Biotec, Bergisch Gladbach, Germany) with anti-bovine CD14 mAb (CAM36A, Washington State University Monoclonal Antibody Center, Pullman, WA, USA), and anti-mouse IgG_1_ MicroBeads (Miltenyi Biotec). CD14^−^ cells were prepared from negative fractions of CD14^+^ cell sorting. The purity of cell populations was confirmed using FACS Verse (BD Biosciences, San Jose, CA, USA). Only highly pure populations (>90%) were used for experiments.

PBMCs, CD14^+^ cells, or CD14^−^ cells (1 × 10^6^ cells) from uninfected cattle were seeded into each well of a 48-well flat-bottom plate (Corning Inc., Corning, NY, USA) with RPMI 1640 medium (Sigma-Aldrich) containing 10% heat-inactivated FBS (Thermo Fisher Scientific), 2 mM L-glutamine, 100 U/ml penicillin, and 100 μg/ml streptomycin (Thermo Fisher Scientific) and were cultured in the presence of live *M. bovis* at a multiplicity of infection (MOI) of 0.1:1, 1:1, or 10:1; 1.5 ng/ml of heat-killed *M. bovis*; 2.5 μM of PGE_2_ (Cayman Chemical, Ann Arbor, MI, USA); or 100 ng/ml of fibroblast-stimulating lipopeptide-1 (FSL-1; Adipogen Life Sciences, San Diego, CA, USA) at 37°C under 5% CO_2_ for 24 h. Heat-killed *M. bovis* was prepared by heating the bacteria to 70°C for 10 min.

To investigate the effects of blocking TLR2 signaling, PBMCs (1 × 10^6^ cells) from uninfected cattle were incubated with sparstolonin B (SsnB; Sigma-Aldrich) in the presence of 100 ng/ml of FSL-1 or 1.5 ng/ml of heat-killed *M. bovis* in a 48-well flat-bottom plate (Corning Inc.) at 37°C under 5% CO_2_ for 24 h. DMSO (Nacalai Tesque, Kyoto, Japan) was used as negative controls.

To assess the effect of soluble factors on PD-L1 expression, 24-h culture supernatants of PBMCs incubated with live *M. bovis* were collected and fresh PBMCs were cultured in these culture supernatants. To prepare 24-h culture supernatants, PBMCs (1 × 10^6^ cells) from uninfected cattle were seeded and were cultured with or without live *M. bovis* at a MOI of 0.1:1, 1:1, or 10:1 as described above. After centrifugation (300 × *g* for 3 min), the culture supernatants of PBMCs were collected. Bacteria and cell-free supernatants were obtained by filtration through a 0.2-μm filter (Pall Life Sciences, Washington, NY, USA). To confirm that the supernatants were not contaminated with *M. bovis*, each supernatant was incubated in NK broth (Miyarisan Pharmaceutical) for 72 h and the broth samples were tested with PCR targeting *M. bovis*-specific gene (21). Then, fresh PBMCs were isolated from the same individual and cultured in the 24-h culture supernatants at 37°C under 5% CO_2_ for 24 h.

### Quantitation of PGE_2_ by ELISA

To investigate whether *M. bovis* antigen and TLR2 signaling promote PGE_2_ production, PBMCs, CD14^+^ cells, or CD14^−^ cells (1 × 10^6^ cells) from uninfected cattle were incubated with live *M. bovis*, heat-killed *M. bovis*, or FSL-1 (Adipogen Life Sciences) as described above. Culture supernatants were collected, and PGE_2_ levels were measured using Prostaglandin E_2_ Express ELISA kit (Cayman Chemical), according to the manufacturer's instructions. The optical density was read at 450 nm in a microplate reader (Corona Electronics, Tokyo, Japan). To compare PGE_2_ levels in peripheral blood between infected cattle and uninfected cattle, PGE_2_ concentrations in serum or plasma were measured using ELISA.

### Flow Cytometric Analysis of PD-L1

To investigate PD-L1 expression on immune cells, PBMCs were blocked with PBS containing 10% goat serum (Thermo Fisher Scientific) at 37°C for 15 min and were washed and stained with anti-bovine PD-L1 mAb (4G12, rat IgG_2a_; 20) or rat IgG_2a_ isotype control (R35-95, BD Biosciences) in the presence of anti-CD11b mAb (CC126, mouse IgG_2b_, Bio-Rad, Hercules, USA) at room temperature for 20 min. After washing with PBS containing 1% BSA (Sigma-Aldrich), cells were stained with PerCp/Cy5.5-conjugated anti-CD14 mAb (CAM36A, mouse IgG_1_, Washington State University Monoclonal Antibody Center), APC-conjugated anti-rat immunoglobulin polyclonal Ab (Southern Biotech, Birmingham, AL, USA), FITC-conjugated anti-mouse IgG_2b_ polyclonal Ab (Beckman Coulter, Fullerton, CA, USA), and Fixable Viability Dye eFluor 780 (eBioscience, San Diego, CA, USA) at room temperature for 20 min. PerCp/Cy5.5-conjugated mouse IgG_1_ isotype control (15H6, Southern Biotech) and mouse IgG_2b_ isotype control (A-1, Southern Biotech) were used as isotype-matched control antibodies for fluorescence minus one staining. mAbs CAM36A and 15H6 were conjugated with PerCp/Cy5.5 using the Lightning-Link PerCp/Cy5.5 Antibody Labeling Kit (Innova Biosciences, Cambridge, England, UK). Cells were then washed and immediately analyzed using FACS Verse (BD Biosciences) and FACSuite Software (BD Biosciences).

### IFN-γ Assay

To investigate IFN-γ levels in peripheral blood of infected cattle, IFN-γ concentration in plasma were measured using Bovine IFN-γ ELISA development kit (Mabtech, Nacka Strand, Sweden). To investigate *M. bovis*-specific IFN-γ response in infected cattle, PBMCs (4 × 10^5^ cells) from infected cattle were incubated with 1.5 μg/ml of heat-killed *M. bovis* in 96-well plates (Corning, Inc.) at 37°C under 5% CO_2_ for 5 days. Subsequently, culture supernatants were obtained from individual wells and were tested for bovine IFN-γ using the ELISA kit (Mabtech) as described above.

### Immunohistochemical Assays of PD-L1 and PGE_2_

Immunohistochemical assays were performed as previously described (18), with slight modification. Briefly, lung tissue was collected from *M. bovis*-infected cattle with pneumonia (Japanese Black, ale, 2 months old). *M. bovis* infection was confirmed with the loop-mediated isothermal amplification assay at Miyazaki University as described previously (22). Then, sections of the lung lesions were immunohistochemically stained for PGE_2_ and PD-L1 using anti-PGE_2_ polyclonal Ab (ab2318, Abcam, Cambridge, England, UK) and anti-PD-L1 mAb (6C11-3A11, Rat IgG_2_a; 22).

### Functional Analysis of Combined COX-2 Inhibition and PD-L1 Blockade

To evaluate T-cell response against the bacterial antigenic stimulation, cell proliferation assay was performed. PBMCs isolated from *M. bovis*-infected cattle were labeled with carboxyfluorescein diacetate succinimidyl ester (CFSE) (Thermo Fisher Scientific) and cultured with 1.5 μg/ml of heat-killed *M. bovis* in 96-well plates (Corning, Inc.) with RPMI 1640 medium (Sigma-Aldrich) supplemented with 10% heat-inactivated FBS (Thermo Fisher Scientific), 2 mM L-glutamine, 100 U/ml penicillin, and 100 μg/ml streptomycin (Thermo Fisher Scientific) at 37°C with 5% CO_2_ for 5 days. Then, PBMCs were harvested and incubated in PBS containing 10% goat serum (Sigma-Aldrich) at room temperature for 15 min to prevent non-specific reactions. The cells were then stained with anti-CD4-Alexa Fluor 647 (CC30; Bio-Rad), anti-CD8-PerCp/Cy5.5 (CC63, Bio-Rad), anti-IgM-PE/Cy7 antibodies (IL-A30; Bio-Rad), and Fixable Viability Dye eFluor 780 (eBioscience, San Diego, CA, USA) at room temperature for 20 min. mAb CC30 was pre-labeled with Alexa Fluor 647 Zenon Mouse IgG_1_ Labeling Kit (Thermo Fisher Scientific). mAbs CC63 and IL-A30 were conjugated with PerCp/Cy5.5 and PE/Cy7, respectively, with Lightning-Link Conjugation Kits (Innova Biosciences). The cells were then washed with PBS containing 1% BSA (Sigma-Aldrich) and analyzed immediately by FACS Verse (BD Biosciences) and FACSuite Software (BD Biosciences).

To investigate the effects of dual blockade using a COX-2 inhibitor and anti-PD-L1 mAb, PBMCs (4 × 10^5^ cells) from infected cattle were incubated with 10 μg/ml of anti-bovine PD-L1 mAb (4G12; 21) and/or 10 μM of meloxicam (Sigma-Aldrich) in the presence of 1.5 μg/ml of heat-killed *M. bovis*, or 1.0 μg/ml of anti-CD3 mAb (MM1A, Washington State University Monoclonal Antibody Center) and 1.0 μg/ml of anti-CD28 mAb (CC220, Bio-Rad) in 96-well plates (Corning, Inc.) with RPMI 1640 medium (Sigma-Aldrich) supplemented with 10% heat-inactivated FBS (Thermo Fisher Scientific), 2 mM L-glutamine, 100 U/ml penicillin, and 100 μg/ml streptomycin (Thermo Fisher Scientific) at 37°C under 5% CO_2_ for 5 days. DMSO (Nacalai Tesque) and rat IgG (Sigma-Aldrich) were used as negative controls. Culture supernatants were obtained from individual wells and were tested for IFN-γ production using the ELISA kit (Mabtech) as described above.

### Statistical Analysis

Differences were identified using Dunnett's test, Mann–Whitney *U*-test, and Steel–Dwass test. Correlation statistics were analyzed using Spearman correlation analysis. The statistical analysis program MEPHAS (http://www.gen-info.osaka-u.ac.jp/MEPHAS/) was used to perform statistical analysis. A *P* < 0.05 was considered statistically significant.

## Results

### *M. bovis* Infection Upregulated PD-L1 Expression via PGE_2_

**Figure 1:**
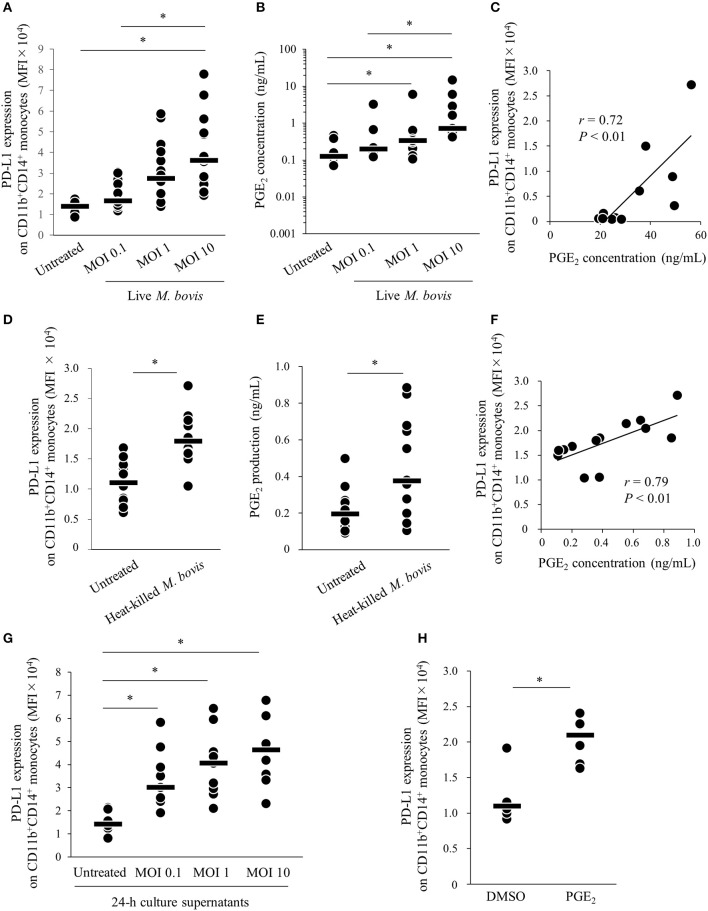
*M. bovis* upregulates PD-L1 and PGE_2_ expression. **(A–F)** PBMCs from *M. bovis*-uninfected cattle were incubated with **(A–C)** live *M. bovis* (MOI of 0.1, 1, or 10) or **(D–F)** heat-killed *M. bovis* (1.5 ng/ml) for 24 h. **(A)** PD-L1 expression on CD11b^+^CD14^+^ monocytes (untreated: *n* = 14, MOI 0.1: *n* = 14, MOI 1: *n* = 14, MOI 10: *n* = 14) was determined using flow cytometry. **(B)** PGE_2_ levels in culture supernatants (untreated: *n* = 9, MOI 0.1: *n* = 9, MOI 1: *n* = 9, MOI 10: *n* = 9) were determined using ELISA. **(C)** Positive correlation is noted between PD-L1 expression on CD11b^+^CD14^+^ monocytes and PGE_2_ levels under live *M. bovis* stimulation (MOI of 10, *n* = 11). **(D)** PD-L1 expression on CD11b^+^CD14^+^ monocytes (untreated: *n* = 13, heat-killed *M. bovis*: *n* = 13) were determined using flow cytometry. **(E)** PGE_2_ levels in culture supernatants (untreated: *n* = 13, heat-killed *M. bovis*: *n* = 13) were determined using ELISA. **(F)** Positive correlation is noted between PD-L1 expression on CD11b^+^CD14^+^ monocytes and PGE_2_ levels under heat-killed *M. bovis* stimulation (*n* = 13). **(G)** PD-L1 expression on CD11b^+^CD14^+^ monocytes cultured in 24-h culture supernatant of PBMCs with or without live *M. bovis* (untreated: *n* = 9, MOI 0.1: *n* = 9, MOI 1: *n* = 9, MOI 10: *n* = 9) were analyzed by flow cytometry. **(H)** PBMCs from uninfected cattle were incubated with PGE_2_, and PD-L1 expression on CD11b^+^CD14^+^ monocytes (DMSO: *n* = 6, PGE_2_: *n* = 6) was determined using flow cytometry. The bars are the median of the values **(A,B,D,E,G,H)**. Statistical significance was determined by the Steel–Dwass test **(A,B,G)** or Mann–Whitney *U*-test **(D,E,H)**. Correlation statistics were analyzed using Spearman's correlation analysis **(C,F)**. *P < 0.05.

Our previous study showed that expression of PD-L1 on monocytes was increased in PBMCs of *M. bovis*-infected cattle (11). To confirm whether *M. bovis* directly upregulates PD-L1 expression in bovine PBMCs during *in vitro* infection, PBMCs from uninfected cattle were cultured with or without live *M. bovis* and PD-L1 expression was analyzed on monocytes by flow cytometry. As shown in Figure S1, CD11b^+^CD14^+^ monocytes were gated and analyzed for expression of PD-L1. PD-L1 expression on CD11b^+^CD14^+^ monocytes was upregulated in the culture with live *M. bovis* when compared with that in the culture without live *M. bovis* (Figure 1A). Our recent studies showed that PGE_2_ was one of the inducers of PD-L1 expression in Johne's disease. Thus, to identify whether PGE_2_ induced by live *M. bovis* upregulated PD-L1 expression, PGE_2_ production in culture supernatants was assessed using ELISA. PGE_2_ production was increased when PBMCs were cultured with live *M. bovis*. Interestingly, PGE_2_ production was positively correlated with PD-L1 expression on CD11b^+^CD14^+^ monocytes (Figures 1B,C). To confirm whether innate immune response against *M. bovis* contribute to PGE_2_ production and PD-L1 expression in the PBMC cultures, PBMCs from uninfected cattle were cultured with or without heat-killed *M. bovis* (Figures 1D–F). Heat-killed *M. bovis* also upregulated PD-L1 expression on CD11b^+^CD14^+^ monocytes in line with increased PGE_2_ production in culture supernatants (Figures 1D–F). To test whether culture supernatants containing PGE2 induced by *M. bovis* upregulated PD-L1 expression, PBMCs from uninfected cattle were cultured in supernatant from PBMCs that were co-cultured with live *M bovis* for 24 h. PD-L1 expression on CD11b^+^CD14^+^ monocytes cultured in 24-h culture supernatants of PBMCs with live *M. bovis* was upregulated when compared with that without live *M. bovis* (Figure 1G). For further confirmation, PBMCs from uninfected cattle were incubated with PGE_2_ and PD-L1 expression on monocytes was analyzed by flow cytometry. PD-L1 expression on CD11b^+^CD14^+^ monocytes incubated with PGE_2_ was increased when compared with that treated with negative controls (Figure 1H). These results indicate that *M. bovis* could upregulate PD-L1 expression on monocytes via PGE_2_.

**Figure 2:**
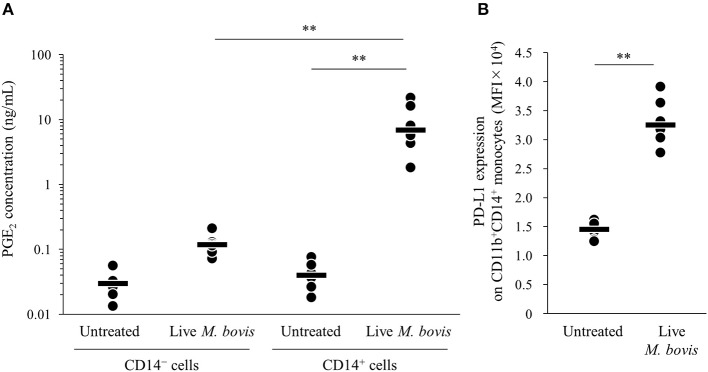
PGE_2_ production from CD14^+^ cells induced by *M. bovis*. CD14^+^ cells or CD14^−^ cells from uninfected cattle were incubated with live *M. bovis* (MOI of 10) for 24 h. **(A)** PGE_2_ production was determined by ELISA (CD14^−^ cells untreated: *n* = 6, CD14^−^ cells Live *M. bovis*: *n* = 6, CD14^+^ cells untreated: *n* = 6, CD14^+^ cells *M. bovis*: *n* = 6). **(B)** PD-L1 expression on CD14^+^ cells (untreated: *n* = 6, live *M. bovis*: *n* = 6) was determined by flow cytometry. The bars are the median of the values **(A,B)**. Statistical significance was determined by the Steel–Dwass test **(A)** or Mann–Whitney *U*-test **(B)**. **P < 0.01.

To identify the major cell type that produced PGE_2_ in the PBMC culture, PGE_2_ productions from isolated CD14^+^ and CD14^−^ cells cultured with live *M. bovis* were measured using ELISA. PGE_2_ production from CD14^+^ cells was significantly increased when compared with that from CD14^−^ cells (Figure 2A). In the CD14^+^ fraction, PD-L1 expression on CD11b^+^CD14^+^ monocytes cultured with live *M. bovis* was increased (Figure 2B). These results represent that CD14^+^ cells could be a major cell type producing PGE_2_ against *M. bovis*.

### Induction of PGE_2_ via Toll-Like Receptor (TLR) 2

**Figure 3:**
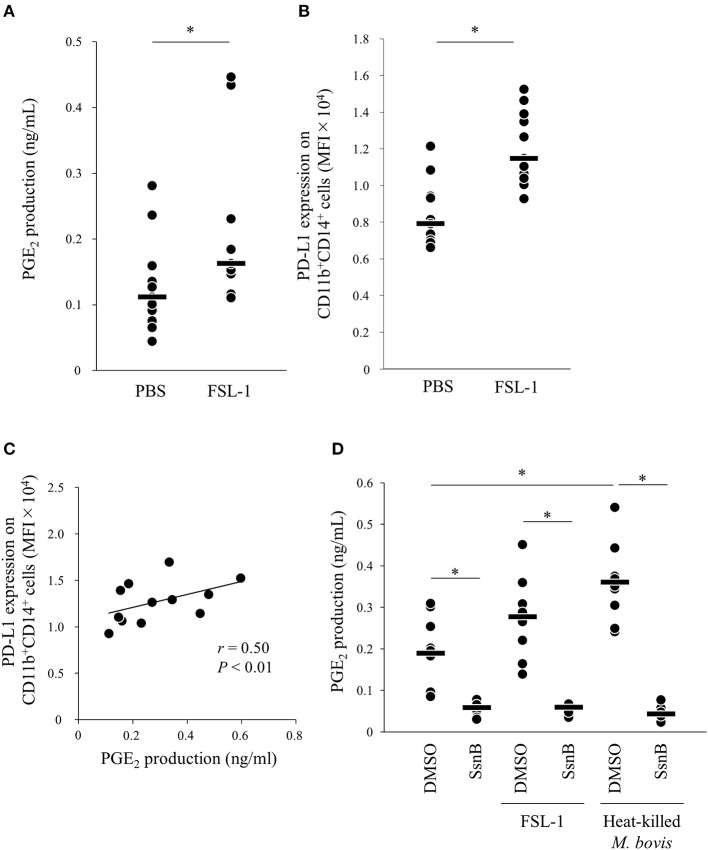
TLR2 signaling upregulates PD-L1 and PGE_2_ expression. PBMCs from uninfected cattle were incubated with FSL-1 (100 ng/ml). **(A)** PGE_2_ production was determined by ELISA (PBS: *n* = 11, FSL-1: *n* = 11). **(B)** PD-L1 expression on CD14^+^ cells (PBS: *n* = 11, FSL-1: *n* = 11) was determined by flow cytometry. **(C)** Positive correlation is noted between PD-L1 expression on CD11b^+^CD14^+^ monocytes and PGE_2_ levels under FSL-1 stimulation (*n* = 13). **(D)** PBMCs from uninfected cattle were incubated with FSL-1 (100 ng/ml) or heat-killed *M. bovis* (1.5 ng/ml) under inhibition of TLR signaling by SsnB and PGE_2_ production was determined by ELISA (DMSO: *n* = 9, SsnB: *n* = 9, DMSO and FSL-1: *n* = 9, SsnB and FSL-1: *n* = 9, DMSO and *M. bovis*: *n* = 9, SsnB and *M. bovis*: *n* = 9). The bars are the median of the values **(A,B,D)**. Statistical significance was determined by the Mann–Whitney *U*-test **(A,B)** or the Steel–Dwass test **(D)**. Correlation statistics were analyzed using Spearman's correlation analysis **(C)**. *P < 0.05.

Because PGE_2_ production was increased by heat-killed *M. bovis*, we hypothesized that pattern-recognition receptors recognized *M. bovis* and their signaling induced PGE_2_ production. Previous research showed that other mycoplasma, *M. fermentans*, which was first isolated from the human urogenital tract (25), induced PGE_2_ production via TLR2 in human monocytes (26, 27). Thus, to test whether PGE_2_ production and PD-L1 upregulation were induced in bovine PBMCs under TLR2 stimulation, PBMCs from uninfected cattle were incubated with a TLR2/6 agonist (FSL-1, 28), which is a synthetic lipoprotein of *M. salivarium*, which been implicated in eye and ear disorders, oral infection, septic arthritis, and periodontal disease in human (28), and PGE_2_ production in culture supernatants and PD-L1 expression on monocytes were analyzed. FSL-1 upregulated PGE_2_ production from PBMCs and PD-L1 expression on CD11b^+^CD14^+^ monocytes (Figures 3A,B). Consistent with the results of *M. bovis* stimulation, PGE_2_ production was positively correlated with PD-L1 expression on CD11b^+^CD14^+^ monocytes under FSL-1 stimulation (Figure 3C). Inhibition of TLR2/4 signaling by a selective antagonist (sparstolonin B; SsnB) decreased PGE_2_ production induced by FSL-1 and *M. bovis* stimulation (Figure 3D). CD14^+^ cells are considered as one of the major cell types expressing TLR2 (29). These results suggest that *M. bovis* could induce PGE_2_ production from monocytes presumably via TLR2.

### Upregulation of PGE_2_ Production in *M. bovis*-Infected Cattle

**Figure 4:**
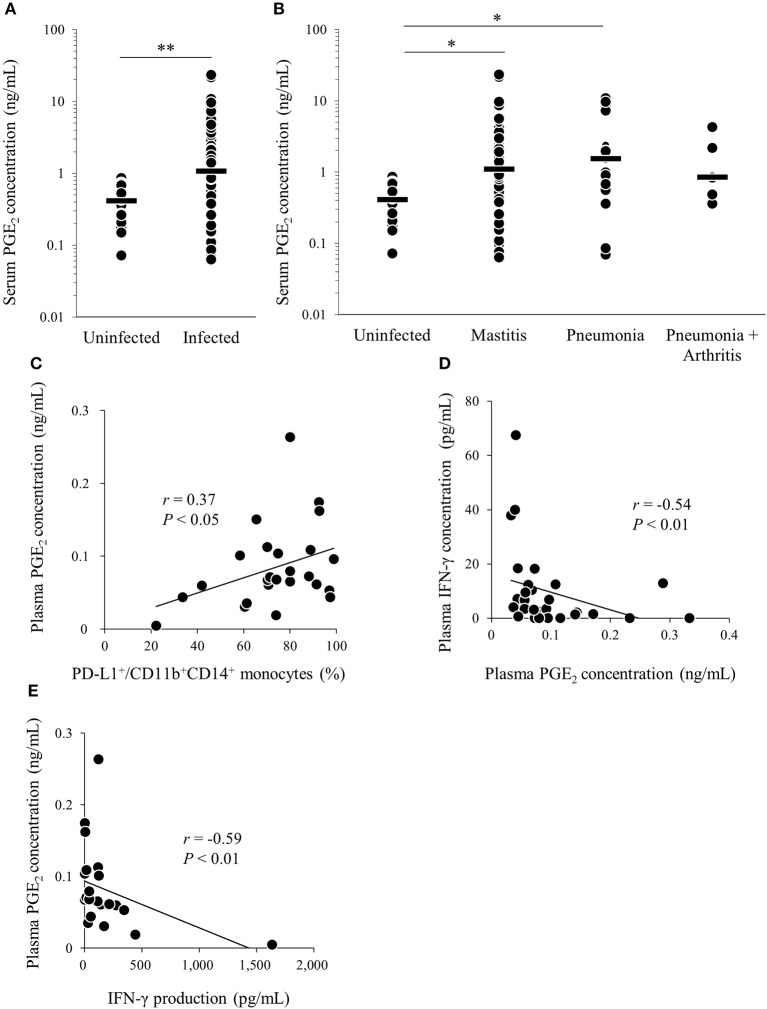
Analysis of PGE_2_ in cattle infected with *M. bovis*. **(A,B)** Serum PGE_2_ levels in *M. bovis*-infected cattle (*n* = 89; Mastitis: *n* = 62, Pneumonia: *n* = 21, Pneumonia with arthritis: *n* = 6) and uninfected cattle (*n* = 18) was determined by ELISA. **(C–E)** Correlation between plasma PGE_2_ levels in *M. bovis*-infected cattle with arthritis (*n* = 13), otitis (*n* = 10), pneumonia (*n* = 5), and other factors. **(C)** Correlation between plasma PGE_2_ levels and plasma IFN-γ levels (*n* = 29). **(D)** Correlation between the plasma levels of PGE_2_ and the proportions of PD-L1^+^ monocytes (*n* = 25). **(E)** Correlation between plasma PGE_2_ levels and IFN-γ production from PBMCs against heat-killed *M. bovis* (*n* = 25). The bars are the median of the values **(A,B)**. Statistical significance was determined by the Mann–Whitney *U*-test **(A)** or the Steel–Dwass test **(B)**. Correlation statistics were analyzed using Spearman's correlation analysis **(C–E)**. *P < 0.05, **P < 0.01.

We found that *M. bovis* induced PGE_2_ production and upregulated PD-L1 expression *in vitro*. To confirm that PGE_2_ is associated with immune dysfunction in bovine mycoplasmosis, we analyzed serum PGE_2_ levels in cattle naturally infected with *M. bovis*, which were diagnosed in the previous studies (21, 30). Serum PGE_2_ levels were significantly increased in *M. bovis*-infected cattle when compared with that in uninfected cattle (Figure 4A). No differences in serum PGE_2_ levels were observed among the mycoplasmosis types with different clinical symptoms in the *M. bovis*-infected cattle (Figure 4B). Because PBMC samples were not available for the previous clinical samples used in Figures 4A,B, we collected blood samples of *M. bovis*-infected cattle and analyzed plasma levels of PGE_2_ and other immunological factors (Figures 4C–E). Interestingly, plasma levels of PGE_2_ were positively correlated with the proportions of PD-L1^+^CD11b^+^CD14^+^ cells in infected cattle (Figure 4C). To investigate the relationship between PGE_2_ and immune response, plasma IFN-γ levels and IFN-γ production against heat-killed *M. bovis* from PBMCs of infected cattle were analyzed using ELISA and their correlation with plasma PGE_2_ levels. Plasma IFN-γ levels and IFN-γ production against *M. bovis* were negatively correlated with plasma PGE_2_ levels in infected cattle (Figures 4D,E). These results suggest that PGE_2_ is associated with PD-L1 expression and immune dysfunction in *M. bovis*-infected cattle.

### Expressions of PD-L1 and PGE_2_ Associated With Pneumonic Lung Lesions in *M. bovis*-Infected Cattle

**Figure 5:**
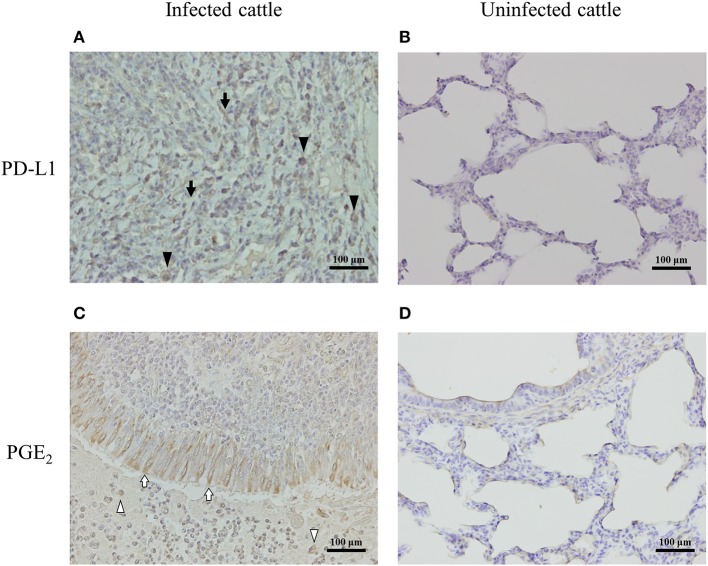
PD-L1 and PGE_2_ expression in the lung of *M. bovis*-infected cattle with pneumonia. Immunohistochemical staining of PD-L1 **(A,B)** and PGE_2_ **(C,D)** in lung tissues of cattle with or without *M. bovis* infection was performed using anti-bovine PD-L1 mAb (6C11-3A11) and human PGE_2_ polyclonal Ab (rabbit polyclonal). Black arrowheads, PD-L1 positive macrophages; black arrows, PD-L1 positive fibroblasts; white arrowheads, PGE_2_-positive macrophages; white arrows, PGE_2_-positive epithelial cells.

To determine the expressions of PD-L1 and PGE_2_ associated with lung lesions in infected cattle, immunohistochemical analysis was conducted on pneumonic lung tissues from cattle with *M. bovis* infection. PD-L1 was expressed on macrophages and fibroblasts infiltrating the lesions (Figure 5A), and PGE_2_ was produced by epithelial cells and macrophages infiltrating the lesions (Figure 5C). On the other hand, the expressions of PD-L1 and PGE_2_ in the healthy lung of *M. bovis*-uninfected cattle were very weak (Figures 5B,D).

### Combined Blockade of the PD-1/PD-L1 Pathway and PGE_2_ Enhanced the *M. bovis* IFN-γ Response

**Figure 6:**
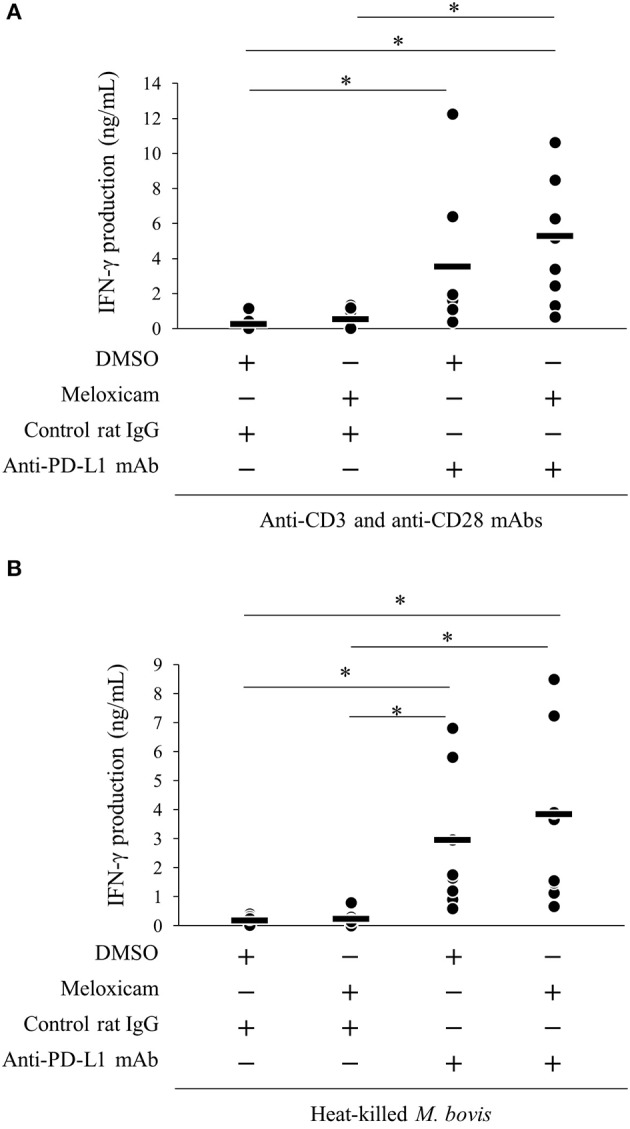
Activation of IFN-γ responses by the combination of meloxicam and anti-PD-L1 Ab. PBMCs from cattle infected with *M. bovis* (*n* = 7) were incubated with meloxicam and anti-PD-L1 mAb in the presence of anti-bovine CD3 and CD28 mAbs **(A)** or heat-killed *M. bovis* **(B)**. IFN-γ production in culture supernatants was determined by ELISA. The bars are the median of the values **(A,B)**. Statistical significance was determined by the Steel–Dwass test **(A,B)**. *P < 0.05.

We previously reported that PD-1/PD-L1 blockade by anti-bovine PD-1/PD-L1 mAbs enhanced the *M. bovis*-specific IFN-γ response *in vitro* (11). Recent research showed that the combined blockade of the PD-1/PD-L1 pathway and PGE_2_ by anti-PD-L1 mAb and a COX-2 inhibitor enhanced immune responses against several pathogens (18, 19, 31). Thus, we evaluated the immune enhancement of dual blockade of the PD-1/PD-L1 pathway and PGE_2_ with regard to the *M. bovis*-specific IFN-γ response using anti-PD-L1 mAb and COX-2 inhibitor, meloxicam. As shown in Figure S2, heat-killed *M. bovis* stimulation induced the proliferative response of CD4^+^ and CD8^+^ T cells in the cultivated PBMCs isolated from *M. bovis*-infected cattle. IFN-γ production from PBMCs stimulated with T-cell stimulators (combination of anti-CD3 mAb and anti-CD28 mAb; Figure 6A) or heat-killed *M. bovis* antigen (Figure 6B) was significantly increased by anti-PD-L1 mAb or the combination of meloxicam and anti-PD-L1 mAb when compared with that in negative controls (combination of DMSO and rat IgG). These results obtained by combining meloxicam and anti-PD-L1 mAb support their potentials as novel treatments for *M. bovis* infection.

## Discussion

Bovine mycoplasmosis, especially associated with *M. bovis* infection, is spreading globally, including in North America, Europe, and Japan (32–36). *M. bovis* is a highly contagious pathogen, and there are no effective vaccines owing to its immunosuppressive effects. Although antibiotics are potentially effective against *M. bovis*, strains resistant to antibiotics have recently been emerging and spreading (37, 38). Therefore, a novel strategy for the control of *M. bovis* infection is required.

Our previous study showed that PD-1-expressing T cells and PD-L1-expressing monocytes were increased in *M. bovis*-infected cattle and that PD-1 and PD-L1 were closely associated with a decreased IFN-γ response against *M. bovis* (11). On the other hand, the mechanism of PD-L1 upregulation during *M. bovis* infection remains unknown. In this study, we revealed that PGE_2_ produced by monocytes was one of the inducers of PD-L1 expression. In addition, we demonstrated that *in vitro* blockade of the PD-1/PD-L1 pathway and PGE_2_ by anti-bovine PD-L1 Ab and a COX-2 inhibitor enhanced the IFN-γ response against *M. bovis*. These results suggest that the PD-1/PD-L1 pathway and PGE_2_ have therapeutic potentials for the control of *M. bovis* infection.

PGE_2_ is typically known as a pro- and anti-inflammatory mediator and its synthesis is induced by COX-2 and PGE synthases (12, 13). Previous research in cancer showed relationships between PGE_2_ and the PD-1/PD-L1 pathway (16, 17). In human melanoma cells, COX-2 was positively correlated with PD-L1 expression (16). In a mouse model of bladder tumor, tumor-infiltrating PD-L1-expressing cells showed high levels of the PGE_2_-forming enzymes, namely, microsomal PGE_2_ synthase 1 (mPGES1) and COX-2 (17). Therefore, PGE_2_ was recently implicated in the induction of PD-L1 expression. Our previous study showed that PGE_2_ upregulated PD-L1 expression on bovine PBMCs and was associated with the progression of Johne's disease, which is a chronic bacterial infection of cattle caused by MAP (18). Then, we hypothesized that this immune dysfunction via PGE_2_-PD-L1 axis is commonly involved in bovine chronic infections. Therefore, this study focused on *M. bovis* infection, which is known to show immune dysfunction induced by exhausted T cell during the disease progression (11). In this study, we demonstrated that *M. bovis* induced PGE_2_ production from monocytes in line with the upregulation of PD-L1 expression on monocytes (Figures 1A–F). Additionally, we showed that the levels of plasma PGE_2_ and proportions of circulating PD-L1^+^ monocytes were positively correlated (Figure 4C). These results indicate that PGE_2_ could be associated with PD-L1 expression in *M. bovis* infection. Several reports showed that PGE_2_ activates STAT3 signaling (18, 39). In tumor studies, STAT3 regulates PD-L1 expression transcriptionally by binding to its promoter (40, 41). These results indicate that PGE_2_ induced by *M. bovis* could upregulate PD-L1 expression on monocytes via STAT3 signaling. On the other hand, many researchers have reported that PD-L1 expression can be induced by other factors, such as IFN-γ and TNF-α production (41, 42). A previous report showed that *M. bovis* induced IFN-γ and TNF-α production from PBMCs of uninfected cattle (30). Further studies on other cytokines are warranted to confirm the mechanisms of PD-L1 expression during *M. bovis* infection.

TLRs are pattern-recognition receptors that play important roles in early innate recognition and host immune responses against several pathogens (43, 44). TLR2 plays the most important role in Mycoplasma infection. TLR2 recognizes lipoproteins from *Mycoplasma* spp. including *M. bovis* (25, 26, 45–48). TLR2/MyD88 signaling induces PGE_2_ production by COX-2 transcription via NF-κB (49), during not only Mycoplasma infection (27, 50, 51) but also other bacterial infection such as *Mycobacterium bovis* (52), *Mycobacterium leprae* (53), and *Staphylococcus aureus* (54). TLR2 expression has been demonstrated in several immune cells, such as lymphocytes, monocytes (28), and some types of non-hematopoietic cells, such as epithelial cells (55). A previous study in humans showed that CD14^+^ monocytes expressed the highest level of TLR2 in blood (56). Indeed, the current study revealed that CD14^+^ monocytes were a major cell type producing PGE_2_ *in vitro* response to *M. bovis* (Figure 2A). In addition, immunohistochemical analysis demonstrated that macrophages infiltrating lung lesions and epithelial cells in lung lesions from infected cattle expressed PGE_2_ (Figure 5B). These findings suggest that *M. bovis* could induce PGE_2_ from monocytes.

TLR4 recognize bacterial lipopolysaccharide and peptidoglycan, which are the components of bacterial cell wall (30, 43). Although Mycoplasmas do not have cell wall (57), several researchers reported the relationships between TLR4 and the inflammation during Mycoplasma infection (55, 58, 59). Shimizu et al. (60) have demonstrated that *M. pneumoniae* induced TNF-α production from macrophages through autophagy and TLR4. This inflammatory response could be need to cytoadherence of live bacteria. Our current study demonstrated that inactivated *M. bovis* and TLR2 stimulation induced PGE_2_ production (Figures 1D, 3A). Inhibition of TLR signaling by SsnB decreased PGE_2_ production induced by FSL-1 and inactivated *M. bovis* (Figure 3D). On the other hand, SsnB selectively blocks not only TLR2- but also TLR4-mediated inflammatory signaling (61). Taken together, although *M. bovis* is recognized via TLR2 and/or TLR4, the results of this study suggest that *M. bovis* could induce PGE_2_ production via mainly TLR2 signaling. However, further studies focusing on TLR4 and *M. bovis* must be needed to elucidate the relationship between *M. bovis* infection and PGE_2_.

PGE_2_ suppresses Th1 immune responses, limiting the functions of natural killer (NK) cells, CD4 T cells, and cytotoxic T cells via specific EP2 and EP4 receptors (14). After binding EP2 or EP4, PGE_2_ induces the expression of anti-inflammatory and immunosuppressive genes by activating the cyclic AMP (cAMP)/protein kinase A/cAMP response element binding protein pathway (14). In the current study, increased plasma PGE_2_ levels were strongly correlated with lower *M. bovis*-specific IFN-γ production (Figure 4D). Additionally, plasma levels of PGE_2_ and proportions of circulating PD-L1^+^CD11b^+^CD14^+^ monocytes were positively correlated (Figure 4C). These results indicate that decreased IFN-γ levels in bovine mycoplasmosis might be associated with the upregulation of PGE_2_ and PD-L1^+^ cells. On the other hand, PGE_2_ can inhibit not only adaptive immunity but also innate immunity. A previous report in humans demonstrated that PGE_2_ inhibited neutrophil functions, such as neutrophil extracellular trap (NET) formation, via EP2 and EP4 (58). Interestingly, in several reports, it is shown that *M. bovis* could inhibit NET formation and escape from neutrophil killing (59). Therefore, PGE_2_ in *M. bovis* infection might play a role as not only an inducer of PD-L1 expression but also a direct suppressor of other immune responses, such as T cell and neutrophil responses. Further studies focusing on inhibition induced by PGE_2_ might help elucidate the mechanisms of immune dysfunction in *M. bovis* infection.

Our previous studies demonstrated that blockade of the PD-1/PD-L1 pathway and/or PGE_2_ effectively improved antigen-specific immune reactions in bovine chronic diseases, such as bovine leukemia virus infection, Johne's disease, and anaplasmosis (18–20, 23, 24, 60, 62, 63, 69). Effective reactivation by dual blockade could be because of PGE_2_ function that inhibition of T-cell activation and regulates PD-L1 expression. Indeed, single blockade of the PD-1/PD-L1 pathway reactivated the *M. bovis*-specific IFN-γ response *in vitro* (Figure 6B). Therefore, we evaluated the immune activation associated with the combined treatment anti-PD-L1 mAb with a COX-2 inhibitor *in vitro*. The combined treatment significantly increased IFN-γ production in response to *M. bovis*. Although a significant difference between single and dual blockade of the PD-1/PD-L1 pathway and PGE_2_ was not observed, IFN-γ production tended to be increased by meloxicam and anti-PD-L1 mAb. To support the efficacy of the combined treatment, further investigation is needed. The combination of PD-1/PD-L1 blockade with COX-2 inhibition has therapeutic potential for controlling *M. bovis* infection.

In other bovine mycoplasmosis, contagious bovine pleuropneumonia caused by *Mycoplasma mycoides* subsp. *mycoides* biotype Small Colony (MmmSC), the number of MmmSC-specific IFN-γ-secreting CD4^+^ T cells was positively correlated with disease recovery (64, 65). IFN-γ promotes the activation and proliferation of T cells and macrophages, which play vital roles in host defenses in the lungs. Previous studies showed that macrophage dysfunction was induced by *Mycoplasma* spp., including *M. bovis*, and promoted persistent infection (2, 66–68). Immune reactivation by combination therapy of anti-PD-L1 mAb with COX-2 inhibitor could help in the development of potential methods for the control of *M. bovis* infection.

Recently, our previous studies reported the establishment of anti-bovine PD-L1 rat-bovine chimeric Ab, and a clinical study on BLV infection was conducted (19, 23). Interestingly, T cell function, which involved IFN-γ response and the proliferation of anti-BLV-specific CD4^+^ T cells, was restored in cattle inoculated with chimeric Ab, while BLV provirus loads were significantly reduced (19, 23). In addition, combined treatment of chimeric Ab with COX-2 inhibitor synergistically decreased BLV provirus loads, clearly demonstrating that this treatment induced antiviral activities (19). However, the *in vivo* effect of combined treatment of chimeric Ab with COX-2 inhibitor in *M. bovis* infection is still unclear and should be determined in further experiments on infected cattle to support the efficacy of this novel treatment approach for clinical application.

In conclusion, the present study found that *M. bovis* can induce PGE_2_ and upregulate PD-L1 expression on monocytes and that *M. bovis*-specific IFN-γ response can be upregulated by dual blockade of the PD-1/PD-L1 pathway and PGE_2_. Thus, PGE_2_ plays an important role in immune dysfunction and is related with the PD-1/PD-L1 pathway. Our findings might contribute to the development of novel strategies for manipulating *M. bovis*-specific T-cell responses to prevent disease progression.

## Data Availability Statement

All datasets generated for this study are included in the article/Supplementary Material.

## Author Contributions

SK, SGot, SM, and KO were responsible for the conception and design of the study. SGot, SK, YH, JK, TO, NM, YSa, KW, EM, AK, RU, SY, MKK, YK, KY, and MTo performed the experiments. SGot, SK, TO, YSu, SM, and KO analyzed the data. SGon, HH, MK, MTa, ET, RU, SY, MKK, YK, KY, and MTo provided intellectual input, field samples, laboratory materials, reagents, and/or analytic tools. SGot and SK wrote the manuscript. SK, TO, SM, and KO contributed to the revision of the manuscript. All authors reviewed and approved the final manuscript.

### Conflict of Interest

KY and MTo are employed by Fuso Pharmaceutical Industries, Ltd. SGot, SK, TO, NM, YSa, YSu, SM, and KO are authors of a patent application covering materials and techniques described in this paper (PCT/JP2018/27041). The remaining authors declare that the research was conducted in the absence of any commercial or financial relationships that could be construed as a potential conflict of interest.
