# Supplementary figures and images for: Upregulation of PD-L1 Expression by Prostaglandin E2 and the Enhancement of IFN-γ by Anti-PD-L1 Antibody Combined With a COX-2 Inhibitor in Mycoplasma bovis Infection
Source: Front Vet Sci. 2020 Feb 20;7:12. doi: 10.3389/fvets.2020.00012 (PMC7045061; doi:10.3389/fvets.2020.00012)

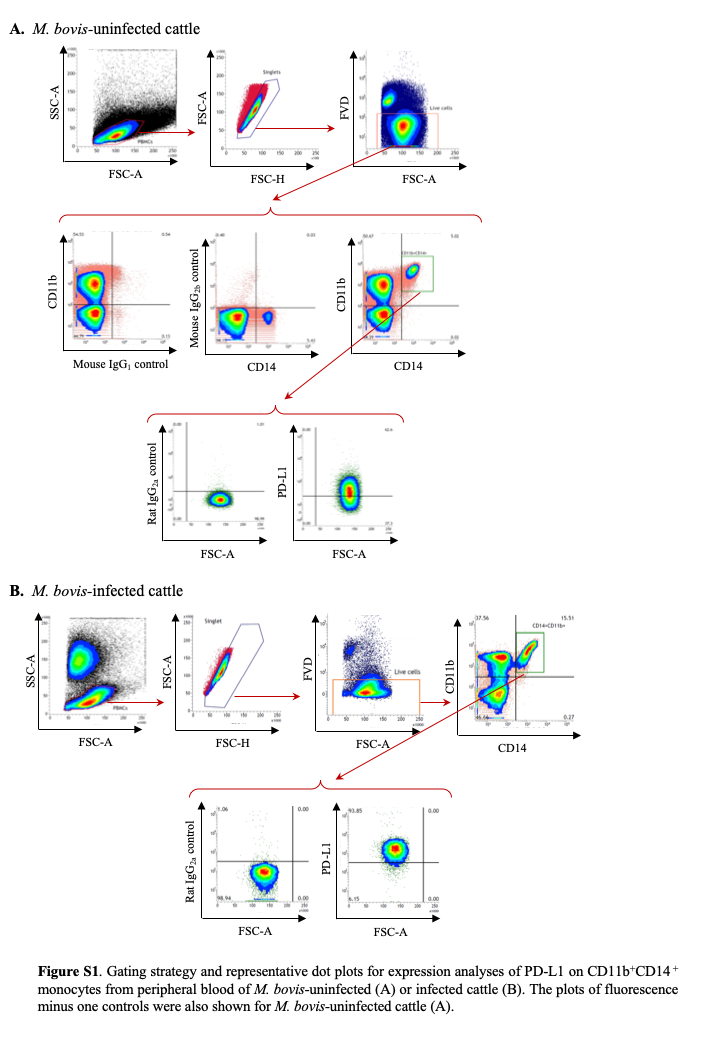

Supplement: Supplementary file 1 [file Image_1.TIFF]

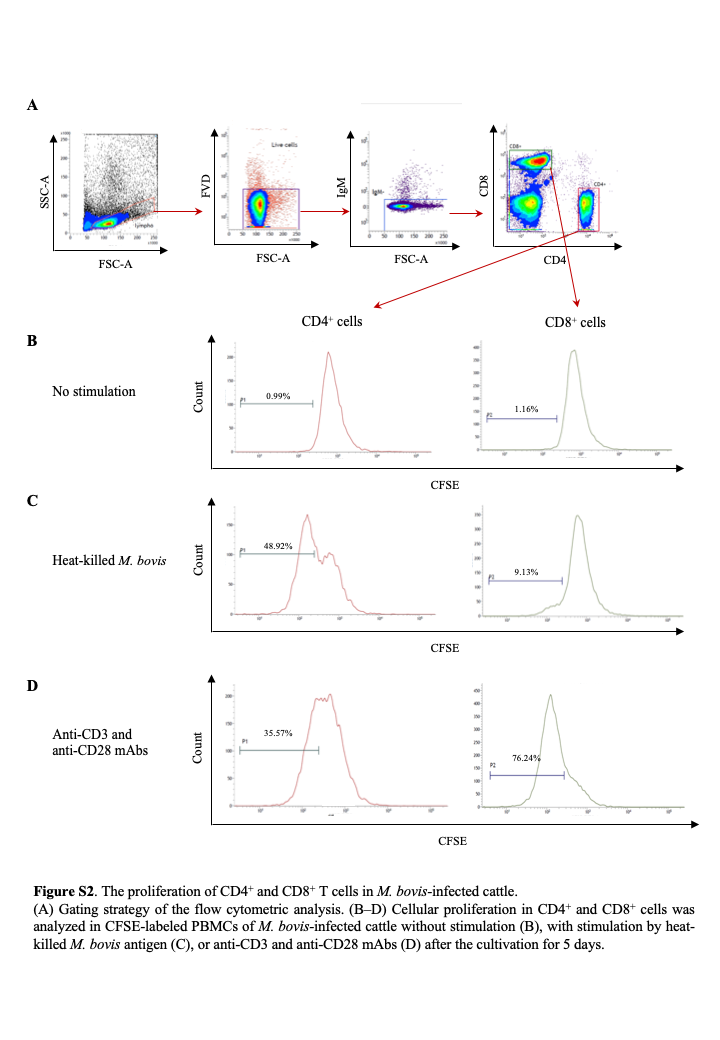

Supplement: Supplementary file 2 [file Image_2.TIFF]
